# Toll‐like receptors in mammalian sperm

**DOI:** 10.1002/rmb2.12651

**Published:** 2025-04-15

**Authors:** Takashi Umehara, Takahiro Yamanaka, Masayuki Shimada

**Affiliations:** ^1^ Graduate School of Integrated Sciences for Life Hiroshima University Higashi‐Hiroshima Japan

**Keywords:** fertilization, sperm, toll‐like receptor

## Abstract

**Background:**

Toll‐like receptors (TLRs) are critical components of the innate immune system and are expressed in various cells, including the reproductive system. Although their roles in female reproductive tissues such as the ovaries and uterus, including their involvement in fertilization and implantation, have been extensively reviewed, their expression and function in male germ cells, particularly in sperm, remain underexplored.

**Methods:**

This review provides a comprehensive summary of research on TLRs expressed in sperm, including findings from experimental models in mice, humans, and industrial livestock.

**Results:**

The activation of TLR2 and TLR4, which detect Gram‐positive and Gram‐negative bacteria, has been shown to reduce sperm motility and viability, thereby impairing fertilization. Conversely, low levels of TLR2 activation have been reported to promote the fertilization of bull sperm, suggesting that TLR2/4 may act as regulators of fertilization. TLR7 and TLR8, which are exclusively expressed in X chromosome‐bearing sperm (X‐sperm), have attracted increasing research interest. These receptors modulate sperm metabolism, selectively reduce the motility of X sperm, and enable the separation of X and Y sperm.

**Conclusion:**

TLRs in the sperm serve as immune receptors that detect bacterial and viral infections, thereby reducing sperm functionality, preventing miscarriage, protecting maternal health, and sex selection.

## 1 INTRODUCTION

Toll‐like receptors (TLRs) are pivotal components of the immune system and are responsible for detecting the pathogen‐associated molecular pattern (PAMP) characteristics of microbial pathogens, such as bacterial lipopolysaccharides (LPSs), viral RNA, and fungal factors. Upon recognizing these molecular patterns, TLRs initiate signaling cascades that activate transcription factors, including nuclear factor‐kappa B (NF‐κB), which regulate genes involved in inflammation and immune responses. This signaling leads to the production of pro‐inflammatory cytokines and type I interferons, which play crucial roles in defense against infections. Beyond their role in pathogen defense, TLR pathways have been implicated in autoimmune and inflammatory diseases, making TLRs a significant focus of immunological research and therapeutic development.^1^, ^2^

The roles of TLRs in reproductive biology have been extensively studied in female reproductive tissues. In the ovary, TLR2 and TLR4 are expressed in cumulus cells surrounding the oocyte during fertilization. Activation of TLR2/4 in cumulus cells induces the secretion of cytokines and chemokines that enhance sperm capacitation and chemotaxis, underscoring their importance in facilitating fertilization.^3^, ^4^, ^5^, ^6^ Interestingly, TLR2 and TLR4 in cumulus cells do not recognize bacteria but are activated by fragmented hyaluronan degraded by hyaluronidase from large molecules. In the uterus, Gram‐negative bacterial LPSs activate TLR4, leading to a robust inflammatory response.^7^, ^8^ Morillo et al. (2020) demonstrated that TLR1/2 is localized to the bovine oviductal epithelium of the ampulla and increases the expression of anti‐inflammatory cytokines in response to sperm.^9^ These findings establish that TLRs are critical regulators of the fertilization process in female reproductive tissues.

On the other hand, TLRs are also expressed in mammalian sperm. Our group demonstrated that TLR2 and TLR4 are localized in the sperm acrosome and that their activation accelerates the acrosome reaction in mice, humans, and boars.^10^, ^11^ In bovine sperm, TLR2 expression is associated with calcium intake and sperm capacitation.^12^, ^13^ Furthermore, TLR7 and TLR8 are exclusively expressed in X chromosome‐bearing sperm (X‐sperm), suggesting their involvement in the functional differences between X and Y sperm through the regulation of metabolic pathways.^14^, ^15^ These findings indicate that TLRs play critical and multifaceted roles in sperm functionality, influencing the journey toward fertilization. This review aims to comprehensively summarize the current knowledge on the involvement of TLRs in sperm function, particularly their roles in mammalian fertilization.

## 2 TLRs

**FIGURE 1:**
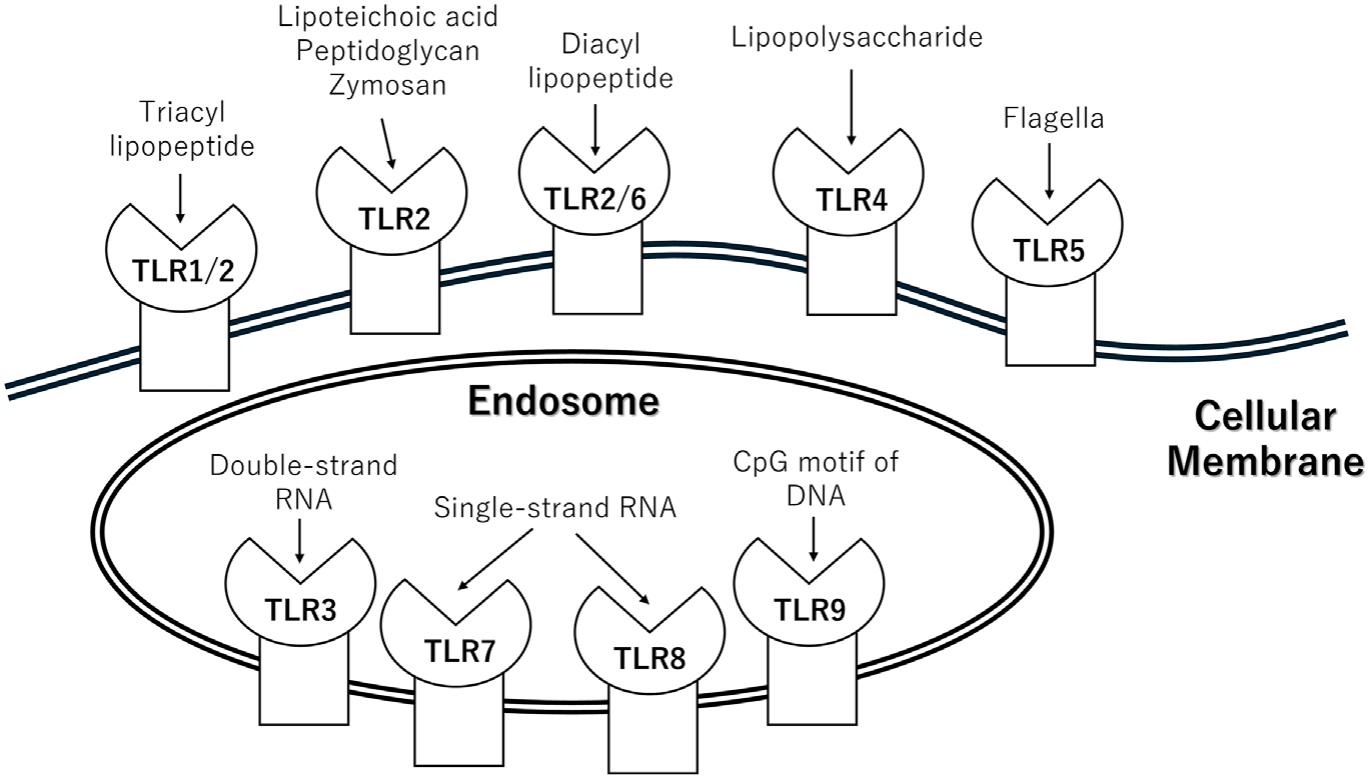
Toll‐like receptor and the agonists. In Toll‐like receptors (TLRs), TLR1, TLR2, TLR4, TLR5, and TLR6 are located on the plasma membrane, while TLR3, TLR7, TLR8, and TLR9 are found on endosomes. TLR1 binds to TLR2 and recognizes triacyl lipopeptides, while TLR6 also interacts with TLR2 to recognize diacyl lipopeptides. TLR2 can form homodimers and recognizes peptidoglycan and Zymosan. TLR4 on the plasma membrane detects lipopolysaccharides (LPS) from Gram‐negative bacteria, and TLR5 is responsible for recognizing flagellin. TLR3, located on endosomes, recognizes double‐stranded RNA; TLR7 and TLR8 recognize single‐stranded RNA, and TLR9 binds to CpG motifs in DNA.

Thirteen types of TLRs have been identified in rodents and 10 in humans, each exhibiting distinct roles and cellular localization^16^ (Figure 1). TLR1, TLR2, TLR4, TLR5, and TLR6 are surface‐expressed receptors predominantly found on immune cells, such as macrophages and dendritic cells, where they interact with extracellular pathogens.^17^ TLR1 forms a heterodimer with TLR2 to detect triacylated lipopeptides, which are abundant in the cell walls of Gram‐positive bacteria. TLR2 recognizes various ligands, including lipoteichoic acid, peptidoglycan, and zymosan and functions as a homodimer or heterodimer with TLR1 or TLR6. TLR4, a well‐known receptor for lipopolysaccharide (LPS), the primary component of Gram‐negative bacterial membranes, requires the co‐receptors MD‐2 and CD14 for effective activation. TLR5 specifically detects flagellin, a structural protein of the bacterial flagella, enabling the identification of motile bacteria. TLR6, in association with TLR2, recognizes diacylated lipopeptides and contributes to the immune response against bacterial lipoproteins, including those from mycoplasmas and certain Gram‐positive bacteria.^16^ These TLRs play a crucial role in the innate immune system by recognizing and responding to pathogens, thus serving as vital components in host defense mechanisms.

TLR3, TLR7, TLR8, and TLR9 are primarily localized in intracellular organelle (endosome) membranes and specialize in the recognition of nucleic acids.^18^ TLR3 is localized in the endosomal membrane, where it detects double‐stranded RNA (dsRNA) from viruses, including members of the *Duplornaviricota* and *Duplopiviricetes* families. TLR7 and TLR8 are present in the membranes of the endoplasmic reticulum and recognize single‐stranded RNA (ssRNA) from viruses such as hepatitis C, Ebola, Zika, and influenza. Severe inflammation, termed a “cytokine storm,” can result from TLR7/8‐dependent activation during infections like COVID‐19, an ssRNA virus that caused a recent global pandemic.^19^ TLR9 detects DNA, particularly the CpG motifs found in viral DNA, within the endoplasmic reticulum. However, TLR9 can also interact with self‐derived DNA, linking it to allergic and autoimmune diseases.^16^ These intracellular TLRs act as primary sensors for nucleic acid‐based pathogens and initiate inflammatory responses against DNA and RNA viruses.

Additional TLRs, such as TLR11,^20^ TLR12,^21^ and TLR13,^22^, ^23^ have been identified in mice. TLR11 is expressed in dendritic and endothelial cells and is activated by protozoan profilin‐like proteins. It forms a heterodimer with TLR12 in an IL‐12‐dependent manner. TLR13 serves as a receptor for the bacterial 23S rRNA and provides an additional layer for pathogen recognition.

These findings highlight the diversity and complexity of TLR‐mediated immune responses in different species.

## 3 LOCALIZATION OF TLRs IN MAMMALIAN SPERM

Mammalian sperm is divided into three regions: the head, midpiece, and tail. The head contains highly condensed DNA that encodes genetic information and is partially covered by the acrosome, which contains enzymes critical for fertilization. The midpiece has the characteristic structure called as “mitochondrial sheath” which mitochondria are coiled in a helical shape. The tail, which is further divided into principal and terminal regions, drives sperm motility. Each region contributes uniquely to successful fertilization.^24^, ^25^

In human sperm, TLR2 has been detected in the acrosome, midpiece, and principal piece of the tail, whereas TLR4 is localized in the acrosome, midpiece, and all parts of the tail.^10^ A similar localization pattern in human sperm for TLR4 was reported by Sahnoun et al. (2017).^26^ Although the localization of TLR2 and TLR4 in mouse sperm has not been reported, their presence has been confirmed by western blotting.^10^ TLR7, TLR8, and TLR9 are also expressed in mouse sperm with distinct localization patterns; TLR7 is found in the principal piece of the tail, TLR8 in the midpiece, and TLR9 in the acrosome and midpiece.^15^, ^27^ Interestingly, the localization of TLR7 in bovine, ram, and goat sperm is similar to that in mouse sperm.^14^, ^28^, ^29^, ^30^

Doğan et al. (2024) investigated the expression and localization of TLR1–5, TLR7, and TLR11–13 in mouse testicular sections via immunohistochemistry.^31^ The results showed that only TLR11 was expressed in spermatogonia, and its expression pattern changed with maturation. In spermatocytes, TLR11, TLR1, TLR3, TLR5, and TLR12 are localized in round spermatids. In addition to these, TLR2, TLR4, TLR7, and TLR13 have been found in elongated spermatids. In the elongated spermatids, the authors distinguished the location of TLR and showed that TLR4, TLR5, TLR11, and TLR13 were localized in the residual body; TLR1, TLR3, TLR5, TLR11, and TLR12 were localized in the endosomal compartment; and TLR1, TLR2, TLR3, TLR5, TLR11, and TLR12 were localized in the acrosome.

Zhu et al. (2016) confirmed the expression of TLR1‐9 (except TLR3) in murine sperm using PCR and evaluated their functionality using TLR agonists.^32^ The effects of agonists such as PamCSK4 (TLR2/1), zymosan (TLR2/6), peptidoglycan (TLR2), LPS (TLR4), flagellin (TLR5), R848 (TLR7/8), and ODN2395 (TLR9) on sperm motility were tested using computer‐assisted sperm analysis (CASA). All ligands, except for the TLR3 agonist poly(I), significantly reduced sperm motility, indicating that TLR1, TLR2, TLR4, TLR5, TLR6, TLR7, TLR8, and TLR9 are functionally active in mouse sperm.

In rat sperm, TLR1, TLR2, TLR3, TLR4, TLR7, TLR8, and TLR9 have been identified,^33^ though their detailed localizations remain underexplored. In humans, the localization of TLR2 and TLR4 has been reported; however, reports on other TLRs are limited. In cattle, TLR2 is localized to the posterior segment of the sperm,^13^ whereas TLR7 and TLR8 are localized in a similar distribution to that observed in mouse sperm.^14^, ^28^ In boars, TLR4 is found in the acrosome and tail,^11^ whereas in goats, TLR7 and TLR8 are present in the midpiece.^29^

**FIGURE 2:**
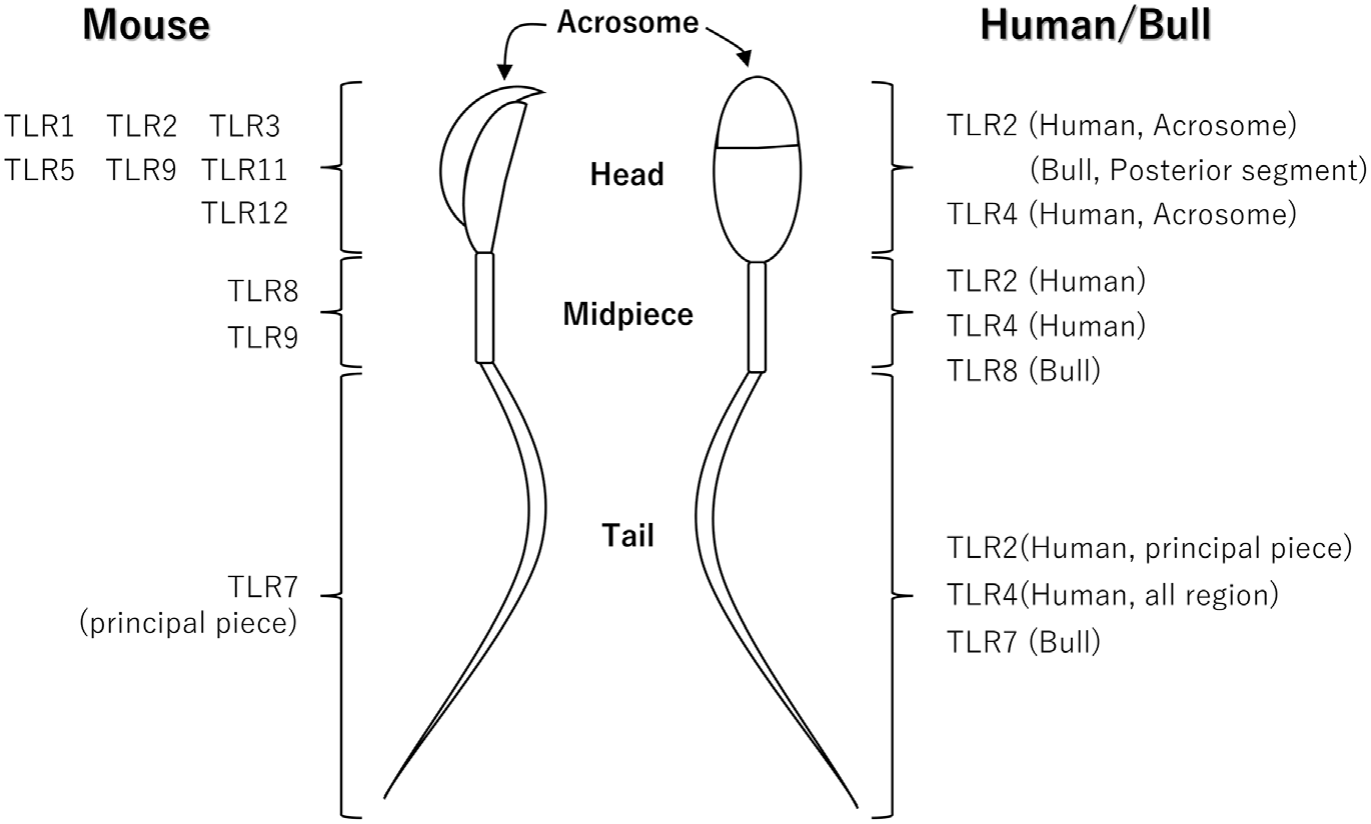
Localization of TLRs in mammalian sperm. In mice, TLR1, TLR2, TLR3, TLR5, TLR9, TLR11, and TLR12 have been reported to localize to the acrosome of the sperm head, TLR8 and TLR9 are localized to the midpiece, and TLR7 is primarily found in the tail, especially in the principal piece. In humans, TLR2 is localized to the acrosome, while both TLR2 and TLR4 are present in the midpiece. Additionally, TLR2 is localized to the principal piece of the tail, and TLR4 is distributed along the entire tail. In bovine sperm, TLR2 is localized to the posterior segment of the head, TLR8 is found in the midpiece, and TLR7 is localized to the tail.

Although the localization and functional roles of TLRs in mammalian sperm remain partially understood, existing evidence suggests that TLRs are expressed across various species and play critical roles in sperm function. Their contribution to sperm motility, fertilization, and pathogen recognition underscores their importance in reproductive biology (Figure 2).

## 4 FUNCTIONS OF TLRs IN MAMMALIAN SPERM

### 4.1 TLR2/4

#### 4.1.1 Infection of gram‐positive and gram‐negative bacteria

Semen infections are widely recognized as a factor that reduces sperm count and impairs sperm quality in humans.^34^, ^35^, ^36^ Sanocka et al. (2005) analyzed the bacterial pattern in the semen of infertile men, and they found the infection of *Staphylococcus haemolyticus* (38%), *Peptostreptococcus* (21%), *Enterococcus faecalis* (20%), *Escherichia coli* (20%), *Ureaplasma urealyticum* (17%), *Mycoplasma hominis* (9%), *Staphylococcus aureus* (7%), and *Bacteroides fragilis* (2%) in the semen.^34^ Most bacteria were Gram‐positive, except for *Escherichia coli* and *Bacteroides fragilis*. Similar findings were observed by Fujita et al. (2010), Fraczek et al. (2014), and Merino et al. (1995),^10^, ^37^, ^38^ indicating that in human semen, infection by both Gram‐positive and Gram‐negative bacteria occurs, and most infected bacteria are Gram‐positive.

In contrast to humans, the bacterial profiles of infected semen from boars showed a predominance of Gram‐negative bacteria. Okazaki et al. (2011) examined semen samples collected from 22 boars and found that *Pseudomonas aeruginosa* was the most common bacterium infecting 13 animals. Other Gram‐negative bacteria, including *Escherichia coli* and *Proteus mirabilis*, were also detected in the three boars. Notably, the only Gram‐positive bacterium identified in this study was *Aerococcus viridans*, which was found in four animals.^11^ Similar studies have corroborated these findings, highlighting the predominance of Gram‐negative bacteria in boar semen infections.^39^, ^40^, ^41^ Therefore, the infection pattern differs between humans and boars, and infection by Gram‐negative bacteria is the main cause of boar semen infection.

#### 4.1.2 Effect of gram‐positive and negative bacteria via TLR2/4

TLR2 primarily recognizes Gram‐positive bacteria, whereas TLR4 specializes in Gram‐negative bacteria.^42^ Studies examining the impact of bacterial infections on sperm quality have utilized TLR agonists, such as Pam3Cys (a TLR2 agonist) and lipopolysaccharide (LPS, a TLR4 agonist) in experimental settings. Treatment of human sperm with these agonists significantly decreased sperm motility and increased DNA fragmentation, as evidenced by TUNEL assay results.^10^, ^43^, ^44^ Conversely, Polymyxin B, a TLR4 antagonist, restores sperm motility and improves DNA integrity in infected semen.^10^, ^45^ Furthermore, a comparative analysis by Sanocka et al. (2005) demonstrated a strong association between infections caused by *Escherichia coli*, *Ureaplasma urealyticum*, and *Staphylococcus aureus* and reduced sperm count and motility.^34^ Collectively, these findings suggest that bacterial infections compromise sperm quality via the TLR2/4 signaling pathway.

The functional importance of TLR2 and TLR4 in the sperm was further elucidated using knockout mouse models. Although TLR2/4 knockout mice were fertile, sperm from wild‐type mice treated with Pam3Cys or LPS exhibited reduced motility and fertilization potential in in vitro fertilization (IVF) experiments. These effects were abolished in sperm from TLR2 or TLR4 knockout mice, highlighting the critical role of these receptors in mediating the negative effects of bacterial infections on sperm function.^10^ Interestingly, in cases of unexplained recurrent spontaneous abortion (URSA) in humans, sperm from affected patients exhibit lower TLR2 and TLR4 expression than sperm from fertile individuals.^46^ This suggests that reduced TLR2/4 expression may mitigate bacterial infection–induced damage to sperm, potentially serving as a protective mechanism in abortion‐prone environments.

Similar effects of TLR activation were observed in porcine sperm. There is a clear negative correlation between the bacterial load in semen and sperm motility with treatments involving Pam3Cys or LPS, which decrease motility and viability.^11^, ^47^, ^48^ The addition of Penicillin G and Polymyxin B to semen extenders improves sperm motility, conception rates, and overall fertility outcomes. Notably, Polymyxin B had a more pronounced effect on frozen‐thawed semen than on fresh semen, as the freezing and thawing processes disrupt Gram‐negative bacterial membranes, releasing LPS and activating TLR4 in sperm. Pretreatment of fresh semen with Penicillin G and Polymyxin B before freezing significantly enhances sperm motility, membrane integrity, and acrosome conditions, leading to marked improvements in conception rates and litter sizes.^49^ These findings highlight the detrimental effects of bacterial infections on semen quality, which are largely mediated by the TLR2/4 pathway.

While most studies have highlighted the negative effects of TLR2/4 activation, recent research has revealed positive roles for TLR2 in bovine sperm. TLR2 was detected in the posterior segment of bovine sperm, and its activation by Pam3Cys increased calcium influx, which is a critical factor for successful fertilization. Conversely, treatment with the TLR2 antagonist CU‐CPT22 suppressed calcium uptake. Activation of TLR2 enhances hyperactivated motility and the acrosome reaction, facilitating penetration of the zona pellucida and ultimately improving fertilization rates in IVF experiments. These findings suggest that TLR2 activation can promote essential processes required for fertilization.^12^, ^13^

The discrepancy between the positive and negative effects of TLR2 activation may be attributed to differences in Pam3Cys concentrations. Human sperm studies typically use a Pam3Cys concentration of ~10 μg/mL^10^; whereas bovine studies employ significantly lower concentrations around 100 ng/mL.^12^, ^13^ This 100‐fold difference highlighted the importance of dose‐dependent responses in TLR‐mediated pathways. Although dose–response studies in human and murine sperm are lacking, the functional impact of TLR2/4 activation likely varies depending on the concentration of agonists or bacterial load across species.

In summary, bacterial infections have profound effects on sperm function and fertility through the TLR2/4 pathway. Although these receptors are often implicated in the deterioration of sperm quality, their activation can support key fertilization processes under specific conditions. Further research is needed to elucidate the context‐dependent roles of TLRs in different species and to develop targeted strategies to mitigate their negative effects while harnessing their potential benefits.

#### 4.1.3 Downstream of TLR2/4 activation in mammalian sperm

**FIGURE 3:**
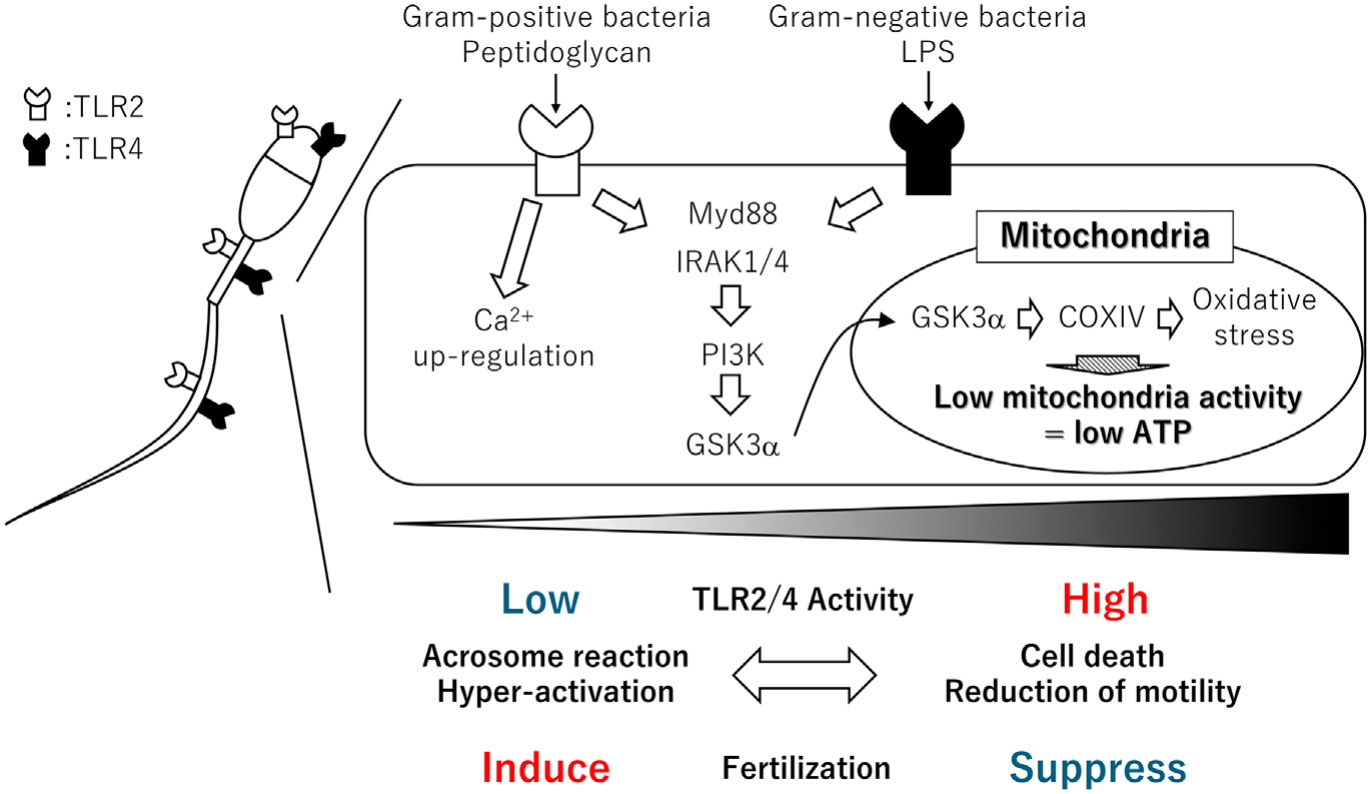
Function of TLR2/4 in mammalian sperm. TLR2/4 expressed in mammalian sperm are localized in the acrosome, midpiece, and tail. When activated by low concentrations of agonists, such as those from Gram‐positive bacteria, TLR2 enhances calcium influx, induces the acrosome reaction, and promotes hyperactivation, all of which are crucial for fertilization. In this context, TLR2 facilitates fertilization. However, when TLR2 is activated at high ligand concentrations, or when lipopolysaccharide (LPS) from Gram‐negative bacteria activates the receptor, mitochondrial oxidative stress is induced, leading to a decrease in ATP production via the activation of Myd88, IRAK1/4, PI3K, and GSK3α. Consequently, sperm death and reduced motility are observed, impairing fertilization.

In immune cells, the activation of TLR2 and TLR4 induces the expression of genes involved in inflammatory responses through the NF‐κB signaling pathway^50^; However, sperm are transcriptionally and translationally inactive due to the high condense of chromatin compaction, which prevents de novo gene expression. Consequently, alterations in sperm quality following TLR2/4 activation are believed to occur through mechanisms that are independent of gene transcription. Zhu et al. (2016) investigated downstream signaling pathways in mammalian sperm following TLR activation using knockout mouse models and specific inhibitors.^32^ Their study demonstrated that TLR activation rapidly triggers the recruitment of adaptor proteins, including MyD88 and IRAK1/4. This activation subsequently led to the phosphorylation of PI3K and GSK3α. Interestingly, GSK3α, initially activated in the cytoplasm, translocated to the mitochondria, where it impaired mitochondrial function, resulting in reduced adenosine triphosphate (ATP) production.^51^, ^52^, ^53^ Because ATP is essential for maintaining sperm motility, its reduction significantly compromises sperm function.^54^, ^55^ A similar effect of TLR4 activation on mitochondrial function was observed in porcine sperm. Treatment with LPS, a TLR4 agonist, decreased sperm motility and increased mitochondrial oxidative stress.^48^ Mitochondrial dysfunction is further characterized by the translocation of mitochondrial transcription factor A (TFAM) from the sperm head to the midpiece, a region enriched in mitochondria. Concurrently, cytochrome c oxidase subunit IV (COXIV) levels were upregulated, indicating enhanced mitochondrial activity. However, the increased activity was accompanied by excessive oxidative stress. Collectively, these findings suggest that the reduction in sperm motility mediated by LPS‐TLR4 signaling, along with the cellular damage caused by bacterial infections, is primarily driven by metabolic disruptions. Specifically, alterations in mitochondrial functionality and the associated increase in oxidative stress play central roles in these processes (Figure 3).

### 4.2 TLR7/8

#### 4.2.1 Infection of virus in the reproductive tract

Viruses can be classified into two categories, DNA and RNA, both of which have been shown to infect reproductive tissues. DNA viruses such as human papillomavirus (HPV) and hepatitis B virus (HBV) have been reported to infect both semen and the uterus.^56^, ^57^, ^58^, ^59^ In addition, several RNA viruses, including human immunodeficiency virus (HIV), hepatitis C virus (HCV), Ebola virus, and Zika virus, have also been identified in semen and the uterus.^60^, ^61^, ^62^, ^63^ Viral infections are not limited to humans but have also been reported in livestock. In cattle, bovine leukemia virus (BLV), which causes bovine leukemia, and bovine herpesvirus 1 (BHV‐1), which is responsible for respiratory and reproductive infections, have been observed to affect reproductive tissues.^64^ Similarly, the porcine reproductive and respiratory syndrome virus (PRRSV) has been linked to reproductive disorders.^65^ These viral infections are recognized by various TLRs, including TLR3, TLR7, TLR8, and TLR9, which play key roles in the immune response to viral pathogens.

#### 4.2.2 Effect of TLR3/7/8/9 activation

Zhu et al. (2016) investigated the response of mouse sperm to toll‐like receptor (TLR) agonists by incubating sperm with poly (I:C), a ligand for TLR3, and R848, a ligand for TLR7/8.^32^ The results showed that no significant effects were observed in poly (I:C)‐treated groups. In contrast, sperm motility was significantly decreased in the R848‐treated group (1 μg/mL), suggesting that TLR3 was nonfunctional, while TLR7/8 was functional in mouse sperm. The authors also measured the intracellular ATP concentrations after R848 treatment and found a significant reduction in ATP levels, which was more pronounced than that observed after treatment with LPS or Zymosan. Furthermore, they observed a decrease in JC‐1 aggregation, a marker of mitochondrial activity, indicating that R848 treatment impaired mitochondrial function in mouse sperm.

Consistent with these findings, our study demonstrated that R848 treatment significantly decreased intracellular ATP levels.^15^ Additionally, R837, a specific agonist of TLR7 (but not TLR8), suppressed ATP concentrations. Interestingly, although R848 treatment reduced JC‐1 aggregation, similar to the findings of Zhu et al. (2016), R837 did not affect JC‐1 aggregation. We also analyzed hexokinase activity, the first enzyme in the glycolytic pathway, and found that it was reduced by both R848 and R837 treatments. This suggests that TLR7 regulates glycolysis in the mouse sperm, whereas TLR8 modulates mitochondrial activity. These findings are consistent with the localization of TLR7 and TLR8 in sperm; TLR7 is predominantly found in the sperm tail, whereas TLR8 is localized in the midpiece. It is well‐established that glycolytic enzymes are primarily localized in the sperm tail, while mitochondria accumulate in the midpiece.^66^, ^67^, ^68^ Regarding TLR7's regulation of glycolysis, R848 treatment increased the phosphorylation of GSK3α/β. GSK3α/β is known to influence hexokinase activity and ATP production, as evidenced by studies showing reduced hexokinase activity and ATP levels in dendritic cells following GSK3α/β inhibition.^69^, ^70^ Furthermore, studies using GSK3α knockout mice have shown infertility due to decreased ATP concentrations, resulting from the disruption of hexokinase phosphorylation.^52^ In conditional GSK3α knockout mice, but not GSK3β knockout mice, hexokinase phosphorylation was significantly reduced in sperm, suggesting that TLR7 may regulate ATP production via GSK3α. These observations in mice have also been reported in cattle, goats, and rams, suggesting that these effects are conserved across mammalian species.^14^, ^28^, ^29^, ^30^

Mihara et al. (2010) investigated the effect of unmethylated CpG DNA (ODN) on sperm by adding ODN to the incubation medium.^27^ The results showed that sperm motility decreased after 6 h of incubation, accompanied by suppression of tyrosine phosphorylation. This reduction in sperm function results in a significant decrease in both in vivo and in vitro fertilization rates. These findings indicate that sperm function is impaired in response to viral pathogen detection by TLR7/8/9 and bacterial pathogen detection by TLR2/4 through metabolic changes.

#### 4.2.3 Challenging the sexing using TLR7/8 activation

In mammals, sex is determined by the pattern of sex chromosomes, specifically X and Y chromosomes. The heterozygous offspring (XY) were male, whereas the homozygous offspring (XX) were female. Each parent produces gametes through meiosis, with the oocyte or female gamete containing only the X chromosome, whereas the sperm or male gamete can carry either the X or Y chromosome. These sex chromosomes differed significantly in size and gene content. For example, the mouse Y chromosome encodes fewer than 700 genes, whereas the X chromosome encodes over 3000 genes.^71^, ^72^ This disparity has also been observed in other mammals, with the DNA content of X‐ and Y‐bearing sperm differing by 3.6% in cattle and 1.3% in pigs.

**FIGURE 4:**
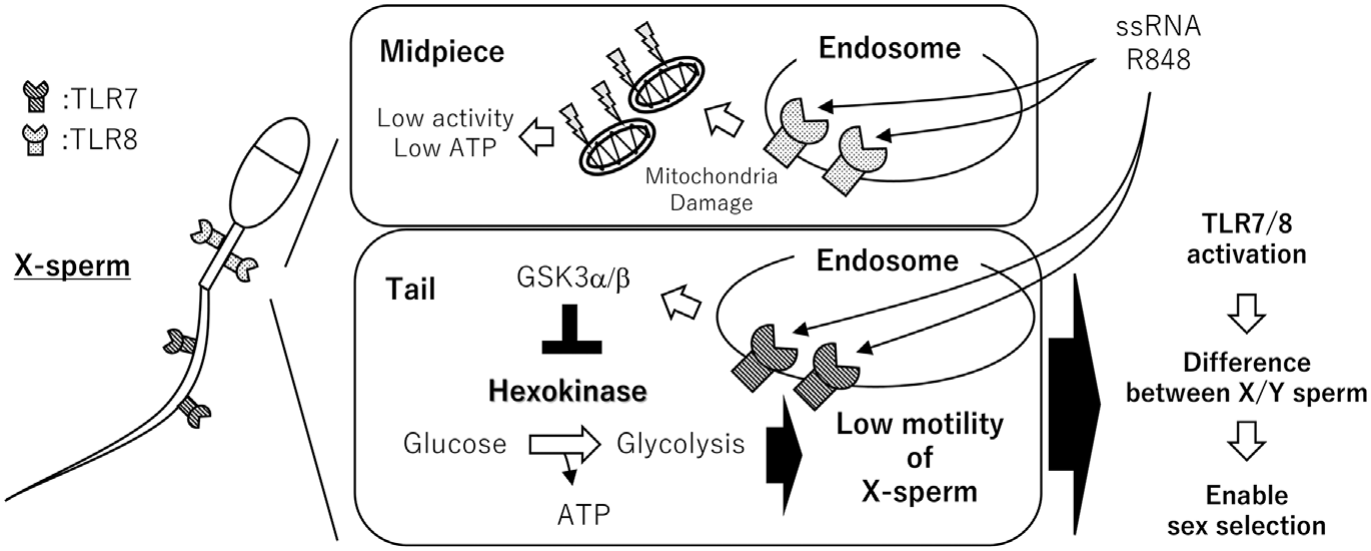
Function of TLR7/8 in mammalian sperm. TLR7/8 expressed in mammalian sperm are activated by single‐strand RNA or the TLR7/8 agonist, R848. TLR8, which is localized in the midpiece, impairs mitochondrial function, while TLR7, expressed in the tail, suppresses the glycolytic pathway. In mice, cattle, and goats, TLR7/8 are exclusively expressed in spermatozoa bearing the X chromosome (X‐sperm). Activation of TLR7/8 specifically reduces the motility of X‐sperm, thereby creating a distinction between X‐ and Y‐sperm. This differential effect enables sex selection.

Exploiting this difference, Johnson et al. employed flow cytometry and Hoechst 33342 staining to successfully separate X‐ and Y‐bearing sperm.^73^ Hoechst 33342 is a DNA‐binding reagent that allows for sperm differentiation based on the DNA content. Sperm stained with this reagent were sorted into high DNA content (X‐sperm) and low DNA content (Y‐sperm) populations using a flow cytometric cell sorter. This technique has been successfully applied to a wide range of mammals including cattle, swine, horses, sheep, goats, dogs, cats, deer, elks, dolphins, water buffaloes, rabbits, mice, and humans^74^ (Figure 4).

In addition to size differences, X and Y chromosomes also exhibit functional disparities. The X chromosome encodes a variety of essential genes, including *G6pdx*, which is involved in glycolysis; *Pdk1*, which participates in the pentose phosphate pathway; and *Ar*, which encodes androgen receptors. Interestingly, while most TLRs are encoded by autosomes, TLR7 and TLR8 are located on the X chromosome. In mice, cattle, and goats, TLR7/8 is expressed in only half of the sperm, but not in horses, and sperm motility is reduced following R848 treatment.^14^, ^15^ This suggests that R848 primarily affects the motility of X chromosome‐bearing sperm (X sperm).

Motile sperm have a natural tendency to swim upward against gravity, making the swim‐up method an effective way to collect them. In a study using the swim‐up method, ~90% of the sperm in the upper layer were Y sperm, while ~80% of the sperm in the lower layer were X sperm. Furthermore, sex selection via in vitro fertilization using separated sperm was successful in over 80% of cases. Similar results were observed in bovine and goat species, reinforcing the idea that TLR7/8 expression is predominantly associated with X‐bearing sperm and that its activation can be used to facilitate sex selection. Recently, there has been increasing interest in refining this technique, with studies exploring sperm selection using double‐stranded DNA and R848.^75^ Our research group is currently working to apply this technology not only in cattle, but also in pigs and dolphins, aiming to enhance its applicability across different mammalian species.

## 5 POTENTIAL APPLICATIONS OF TLRs

Sperm TLR2/4 can be activated by Gram‐positive and Gram‐negative bacterial infections in the semen, leading to a significant decline in sperm function and quality. Since bacterial contamination in semen activates TLR2/4 and impairs sperm function, removing bacteria and/or suppressing TLR activity is crucial for maintaining sperm quality. In ART, Percoll density gradient centrifugation, the swim‐up method, and microfluidics‐based sperm selection are used to collect highly motile sperm and can also eliminate bacteria from semen. Additionally, TLR antagonists have been developed to suppress TLR activity in infected semen.

### 5.1 Percoll density gradient centrifugation

Percoll density gradient centrifugation is widely used in ART to select viable sperm and significantly reduce bacterial contamination, thereby potentially minimizing TLR activation in purified sperm. Kaneko et al. (1986) used a 40%–80% Percoll solution for sperm preparation and reported a marked improvement in progressive motility and viability.^76^ Furthermore, semen samples initially contaminated with *Pseudomonas aeruginosa*, *Acinetobacter calcoaceticus*, *Pseudomonas* species, *Staphylococcus epidermidis*, and *Corynebacterium* species exhibited contamination levels below 0.02% of the original levels after Percoll density gradient centrifugation. Numerous studies demonstrated that this method effectively removes bacteria from semen, making it a suitable approach for ART.

### 5.2 Swim‐up method

The Swim‐up method relies on sperm motility, which allows highly motile sperm to migrate against gravity into the upper layer of the medium.^77^ This procedure helps eliminate some bacteria and debris, which tend to remain in the lower fraction. Sun et al. (1987) compared techniques for selecting bacteria‐free sperm and found that the swim‐up method yielded a higher number of collected sperm than the Percoll gradient approach. However, while bacterial contamination decreased slightly compared with raw semen (8.66 × 10^3^ vs. 6.40 × 10^3^ CFU/mL), Percoll density gradient treatment reduced contamination more substantially, to 0.01 × 10^3^ CFU/mL.^78^ Thus, although the Swim‐up method enriches motile sperm and partially reduces bacterial infection, it is less effective for bacterial removal than Percoll density gradient centrifugation.

### 5.3 Microfluidic‐based sperm selection

Microfluidic devices utilize microscale flow channels and specialized structures to collect motile sperm without extensive centrifugation or multiple handling steps.^79^ This technology is used to reduce bacterial contamination. For example, Jeon et al. (2022) employed a multi‐dimensional double‐spiral inertial microfluidic platform to isolate sperm from 10 μm beads, which served as proxies for cells of similar size. Their findings suggest that microorganisms ~10 μm in diameter could be removed from infected semen using this approach.^80^ Other platforms selectively collect motile sperm using specific flow velocities, physical barriers, or filters combined with the Swim‐up method.^79^ Although direct evidence for bacterial removal in these systems is still limited, the ability to recover only sperm with proper shape and motility suggests that microfluidics‐based selection could effectively reduce bacterial contamination while preserving highly motile sperm. Several commercial devices that employ these principles are now available, potentially offering more convenient and efficient sperm separation.

### 5.4 TLR4 antagonist treatment

Fujita et al. (2007) demonstrated that the addition of Polymyxin B, a TLR4 antagonist, to infected semen suppressed TLR4 activation. Since bacterial infection can compromise sperm function through TLR activation, antagonists such as Polymyxin B help to maintain sperm quality during storage and processing.^10^ Previous studies have reported the beneficial effects of long‐term storage on sperm motility during long‐term storage.^81^, ^82^ This approach has also been applied in livestock production: we previously demonstrated that treating boar semen with Polymyxin B and Penicillin G improved fertilization in bacterially contaminated semen,^49^ indicating that bacterial contamination may reduce fertilization efficiency—possibly via TLR activation—and that targeted interventions can mitigate such effects. These findings suggest that reducing bacteria‐induced TLR activation, either through bacterial removal (e.g., Percoll) or pharmacological inhibition, could enhance ART and livestock production outcomes.

## 6 CONCLUSION

Mammalian sperm express multiple TLRs, which serve as sensors for detecting bacterial infections and play a critical role in safeguarding maternal health by reducing the risk of miscarriage. Additionally, as X chromosome‐specific genes, TLR7 and TLR8 are exclusively expressed in X‐bearing sperm (X‐sperm) and have been implicated in the separation of X sperm from Y sperm. It has been reported that the sex ratio in couples infected with the Zika virus is skewed, suggesting that this phenomenon may occur not only in vitro but also in response to physiological infections. While some functions of TLRs in sperm have been well characterized, others remain unexplored, and the underlying mechanisms are yet to be fully elucidated.

## CONFLICT OF INTEREST STATEMENT

The authors declare no conflict of interest. “Masayuki Shimada” is an Editorial Board member of Reproductive Medicine and Biology and a coauthor of this article.

## HUMAN AND ANIMAL RIGHTS

This article does not include any studies involving human or animal participants.
